# Academical Proceedings, National and Foreign

**Published:** 1822-03

**Authors:** 


### ACADEMICAL PROCEEDINGS,

### NATIONAL AND FOREIGN.

#### 1. ROYAL SOCIETY OF LONDON.

THE meetings of this Society, that have intervened since our last report, have been chiefly taken up with mathematical papers, and the reading of a memoir, which was concluded on Thursday the 21st instant, by the Rev. Mr. BUCKLAND, on the Bones of Antediluvian Animals, discovered in the hyæna's den at Kirkdale, near Kirby Moorside, in Yorkshire. The description given by the author of the place in question is as singular, as the explanation he has offered for the curious fact of bones of the larger animals being found in England is ingenious. The hyæna's den has, from this circumstance, acquired a high degree of celebrity, that has been enhanced by the *jeu d'esprit* of Mr. C.; who, under a lithographic representation of the exterior of the cave, through which the worthy professor is seen to emerge, to the astonishment of four famished hyænas which surround the entrance, has inscribed a poetical account of the professor's memoir, with, among others, the following stanza:—

> "But, of all the miraculous caves,
> And of all their miraculous stories,
> Kirby hole all its brethren outbraves,
> With Buckland to tell of its stories."

#### 2. ROYAL COLLEGE OF SURGEONS OF LONDON.

Sir EVERARD HOME, Bart. serjeant-surgeon to the King, and master of the College, delivered the Hunterian Oration on the 14th of February, before a crowded audience of professional and other characters, who had been invited for the occasion.

#### 3. HUNTERIAN SOCIETY OF LONDON.

On the 6th of February last was held the third anniversary meeting of the Hunterian Society, when the following members were elected for the ensuing year:

*President*—Benjamin Robinson, M.D.

*Vice-Presidents*—William Babington, M.D. F.R.S. B. C. Pierce, M.D. Thomas Callaway, Esq. and John Dunston, Esq.

*Treasurer*—B. Robinson, M.D.

*Secretaries*—J. T. Conquest, M.D. and William Cooke, Esq.

*Council*—Sir William Blizard, F.R.S. H. Greenwood, Esq. Z.

Newington, Esq. J. C. Knight, Esq. R. Dunglison, Esq. J. Miles, Esq. J. Roberts, Esq. Eusebius A. Lloyd, Esq. M. Gosset, Esq. H. Hawkins, Esq. H. Johnson, Esq. and J. Leadham, Esq.

And, on the following day, the members and friends of the Society partook of an excellent dinner at the London Tavern; on which occasion Dr. Babington took the chair, in consequence of the unavoidable absence of the president.

### 4. FACULTY OF MEDICINE OF PARIS.

The professors held their public annual meeting, in November last, for the distribution of prizes to the *elèves* of the Ecole Pratique, the *elèves sage-femmes*, and to the members of the Society of Medical Instruction, founded by the late Baron CORVISART.

The meeting was attended by Baron CUVIER, president of the Royal Council of Public Instruction, in the chair, and by two counsellors and two inspectors general.

After the discourse pronounced by the president of the Faculty and by Baron CUVIER, Professor ORFILA, as secretary, proceeded to read the names of the successful candidates in the following classes:— Anatomy and Pathology, Medical Physic and Chemistry, External Clinic and Operations. Internal Clinic and Action of Medicines.

### 5. MILITARY HOSPITAL OF INSTRUCTION AT THE VAL DE GRACE IN PARIS.

The distribution of prizes to the pupils of the school attached to this hospital took place before Baron JOINVILLE, military intendant of the first division, representing his Excellency the Minister at War. The members of the Army Medical Board, and the principal medical officers belonging to the various military establishments of Paris, were also present.

Mr. LALIBERT, *pharmacien en chef*, pronounced the eulogium of Mr. BRUGMAN, professor of natural history and chemistry in the university of Leyden, who had distinguished himself for the eminent services he had rendered to the French armies during the late eventful war.

The successful candidates in military surgery were four; and two in military pharmacy.

☞ The Royal Academy of Sciences of Berlin have named Professor OERSTED, the Danish philosopher, to whom we are indebted for the late discoveries on magnetism and galvanism, one of the corresponding members in the class of natural philosophy.

☞ Professor KASTNER moves from the university of Bonn on the Rhine to Erlangen, in the capacity of professor of medicine and chemistry, with the title of Royal Bavarian Counsellor.

☞ The professor of therapeia and clinical medicine, in the university of Wilna, Dr. JOSEPH FRANK, son of the late celebrated PETER FRANK of Vienna, has received the order of knighthood of St. Ann, and has been promoted to the rank of counsellor of state.

☞ His Majesty the KING of PRUSSIA has been pleased to confer, on Sir ALEXANDER CRICHTON, Bart. the cross of the Red Eagle of the second class.

